# A Cotton Annexin Protein AnxGb6 Regulates Fiber Elongation through Its Interaction with Actin 1

**DOI:** 10.1371/journal.pone.0066160

**Published:** 2013-06-04

**Authors:** Yiqun Huang, Jin Wang, Lida Zhang, Kaijing Zuo

**Affiliations:** 1 Plant Biotechnology Research Center, SJTU-Cornell Institute of Sustainable Agriculture and Biotechnology, School of Agriculture and Biology, Shanghai Jiao Tong University, Shanghai, China; 2 Biotechnology Research Institute, Chinese Academy of Agricultural Sciences, Beijing, China; University of Toronto, Canada

## Abstract

Annexins are assumed to be involved in regulating cotton fiber elongation, but direct evidence remains to be presented. Here we cloned six Annexin genes (*AnxGb*) abundantly expressed in fiber from sea-island cotton (*G. barbadense*). qRT-PCR results indicated that all six *G. barbadense* annexin genes were expressed in elongating cotton fibers, while only the expression of *AnxGb6* was cotton fiber-specific. Yeast two hybridization and BiFC analysis revealed that AnxGb6 homodimer interacted with a cotton fiber specific actin GbAct1. Ectopic-expressed *AnxGb6* in *Arabidopsis* enhanced its root elongation without increasing the root cell number. Ectopic *AnxGb6* expression resulted in more F-actin accumulation in the basal part of the root cell elongation zone. Analysis of *AnxGb6* expression in three cotton genotypes with different fiber length confirmed that *AnxGb6* expression was correlated to cotton fiber length, especially fiber elongation rate. Our results demonstrated that AnxGb6 was important for fiber elongation by potentially providing a domain for F-actin organization.

## Introduction

Cotton is the chief source of natural fiber for the textile industry. Fiber length is an important agronomic trait in cotton, and considerable attention has been focused on deciphering the molecular mechanisms of fiber elongation [1]. Cotton fiber is a single cell trichome that elongates from the seed coat epidermal cell. Development of cotton fiber can be divided into four distinct and overlapping stages including fiber initiation, fiber elongation, secondary cell wall biosynthesis, and fiber maturation [2], [3]. Extension of cotton fiber cells starts on 0 DPA (day post anthesis) and lasts for about 20 days. The growth of fiber cells reaches a length of 3–5 cm before fiber maturation. The length of fiber cells is mostly determined by their growth rate and mode during elongation stage.

Recent studies indicate that fiber cells probably expand via a linear-growth mode in combination with tip-growth [1]. Various studies also support the theory that linear-growth mode normally includes a high Ca^2+^ gradient formation and cytoskeleton rearrangement in the quick-expanding fiber cell [4]. The staining of cellular Ca^2+^ revealed that fiber initials had more Ca^2+^ than other ovule cells [5]. Calcium signal transduction genes like *CIPK, CaM and GhCPK1* are involved in fiber elongation [5]–[7]. Their transcription levels are significantly lower in the fiber length mutant *li* in comparison with the wild-type [6]. Fiber elongation is also accompanied by active changes in the organization of microtubules and actin cytoskeleton [8], [9]. Among the cotton actin gene family, *GhAct1* is predominantly expressed in cotton fibers. Suppression of *GhAct1* expression dramatically reduces the number of actin bundles, affects fiber cell elongation and shortens fiber length [10]. These results suggest that *GhAct1* plays a major role in fiber elongation. The dynamic rearrangement of actin filaments maintaining a proper balance between filamentous and monomeric actin is possibly the key factor for fiber elongation [10].

The dynamic rearrangement of the actin is controlled by a number of actin-binding proteins like profilin and actin depolymerizing factor ADF [11]–[15]. A cotton profilin *GhPFN2* is expressed in the early stage of fiber elongation. The over-expression of *GhPFN2* caused pre-terminated cell elongation, resulting in obvious decrease in the length of mature fibers. In contrast, increased fiber length and strength was observed in *GhADF1* RNAi plants as compared with the wild-type plants [13], [14]. In the last decade, biochemical evidence has validated that ADF, profilin, and other actin-binding proteins are likely candidates for capping and severing activities; and that upon stimulation by Ca^2+^, these proteins alter the dynamics of actin filaments in root hairs [16]. Transcriptomic and proteomic studies indicate that actin and actin binding proteins are regulated by a Ca^2+^ gradient; however, currently, there is not enough evidence to link actins to the calcium signal transduction pathway proteins during fiber elongation.

The annexins are a multigene family of calcium-dependent or independent membrane phospholipids and cytoskeleton binding proteins, which are widespread in most eukaryotic cells [17]–[20]. Plant annexins are abundant proteins that could comprise 0.1% of the plant cell protein, and exist in the cell wall as well as the cytoplasm [21], [22]. Due to their capability to bind with calcium and lipid membranes, annexins can participate in signaling networks and membrane trafficking [23], [24], including secretion, signal transduction, construction of ion channels, and cytoskeletal interactions [25]–[29]. Plant annexins are concentrated in the expanding tip region of polarly growing cells, such as pollen tubes and root hairs [30]–[32] and their localization corresponds with the directionality of secretion. The expression and accurate localization of annexins can regulate cell polar expansion [33].

In cotton, the studies on annexin proteins have drawn considerable attention because of their role in fiber expansion and their binding capability to calcium and lipid membranes [34]. Rapidly elongating fiber cells contain three to five times the amount of fatty acids (from C20 to C26, mainly sphingolipids) as compared to the ovules [35]. Sphingolipids can stimulate vesicle transport and fiber elongation [36], [37]. In the tip zones of expanding fiber cells, high levels of Ca^2+^, ROS, and even secretory vesicles have been observed, and this is consistent with previous analyses of annexin functions [1], [7], [38]. The up-regulation of *GhFAnnx* and *GhAnx1* during fiber elongation indicates the involvement of these genes in cotton fiber elongation [39], [40]. Four annexin protein iso-variants are markedly down-regulated in the fiber length mutant *li* [41]. Furthermore, ectopic expression of a mustard annexin gene *AnnBj1* in cotton enhances abiotic stress tolerance and fiber quality under stress [42]. These studies led us to hypothesize that annexins might regulate fiber elongation in coordination with actin. Since annexins are a multigene family of proteins, we conducted the present study to determine whether one or several annexins together participate in the regulation of fiber elongation; and whether annexins could directly interact with actin to regulate its remodeling.

In this study, we demonstrated that a fiber-predominantly-expressed gene *AnxGb6* influenced the cotton fiber elongation rate during fiber polar expansion. AnxGb6 was also found to directly interact with F-actin to regulate the mode of the actin assembly.

## Materials and Methods

### Plant materials

Cotton (*Gossypium barbadense* L. cv. Pima-90, *Gossypium hirsutum* L. cv. XU142 and its mutant XU142 fl, Coker 312, T586) were grown in the greenhouse at the Shanghai Jiao Tong University. When cotton plants had grown for about 100 days, the cotton roots, stems, leaves and ovules at different stages were collected and immediately frozen in liquid nitrogen for RNA and DNA extraction. Cotton ovule developmental stage was classified according to the method reported by Hasenfratz and Lee [43], [44]. Wild-type and transgenic *Arabidopsis thaliana* plants (ecotype Columbia, Col-0) were grown in the greenhouse under long-day conditions (22°C, 16/8 h light/dark).

### Total RNA and genomic DNA extraction

Total RNA of cotton tissues was extracted according to the cetyl trimethylammonium bromide (CTAB) extraction method [45]. DNase I (Tiangen, Shanghai, China) was added to remove genomic DNA. In order to eliminate phenol and polysaccharide in total RNA, the RNAprep Plant RNA Purification Kit (Tiangen, Shanghai, China) were used to purify total RNA. *Arabidopsis* total RNA was extracted using the RNAprep Plant RNA Purification Kit (Tiangen, Shanghai, China). The genomic DNA from cotton and *Arabidopsis* were isolated according to the method described by Paterson [46]. The concentration of the purified RNA and DNA was analyzed by a nucleic acid analyzer (DU-640, Beckman).

### Isolation and sequence analysis of *G. barbadense* annexin gene family

Blast analysis was used to find all putative expressed sequence tags (ESTs) for annexin from *G. barbadense* L. fiber ESTs library. The first-strand cDNA was synthesized with 2 µg of total RNA from 0–8 DPA cotton ovules using AMV Reverse Transcriptase (Takara, Japan). The synthesized 1^st^ strand cDNA was then used as the reverse transcript polymerase chain reaction (RT-PCR) template. Based on the obtained EST fragment, gene-specific primers were designed and used for the 3′ and 5′ RACE according to the User Manual of SMART RACE cDNA Amplification Kit (Clontech, USA). The open reading frame cDNAs of annexin genes were obtained by PCR amplification with the primers (Table S1). The 30 µL PCR volume contained 10ng 1^st^ cDNA, 1U ExTaq, 10 pM dNTPs, 5pM MgCl_2_, 10pM primers. PCR amplification was carried out as follows: 94°C for 3 min, followed by 32 cycles of amplification (94°C for 30 sec, 55°C for 30 sec, and 72°C for 3 min) and finally by extension at 72°C for 10 min. The amplified products were purified and cloned into pMD18-T vector (TaKaRa, Japan) and sequenced.

The putative amino sequences of cotton annexin genes were found online by using the open-reading frame (ORF) finder (http://www.ncbi.nlm.nih.gov/). The cotton annexin proteins were aligned with 18 annexins from different organisms by using DNAMAN and ClustalX 1.83 (AnxGh1: AAR13288.1; AnxGh2: AAB67994.1; AnxGhF: AAC33305.1; AnxGhFx: FJ415173; AnxAt1: NP_174810.1; AnxAt2: NP_201307.1; AnxAt3: NP_181410.1; AnxAt4: NP_181409.1; AnxAt5: NP_564920.1; AnxAt6: NP_196584.1; AnxAt7: NP_196585.1; AnxAt8: NP_568271.2; AnxZm2: NP_001105475.1; AnxZm4: NP_001147343.1; AnxZmF: ACF82214.1; AnxZm33: NP_001105728.1; AnxOs1: NP_001061839.1; AnxOs2: NP_001048149.1; AnxOs33: NP_001057176.1). Molecular weight, isoelectric point, functional domains, and amino acid signal peptides of cotton annexins were calculated using the ExPASy online servers (http://cn.expasy.org/tools). A Neighbor-Joining tree of annexin proteins was constructed using MEGA 3.1 program [47].

### Expression pattern analysis of cotton annexin gene family

The real-time quantitative PCR (qRT-PCR) analysis was performed according to the manual of SYBR premix Ex-Taq (Takara, Japan) in a DNA Engine Option 3 System (MJ Research, USA). The 30 µL PCR volume contained 500 ng of 1^st^ strand cDNA, 1U ExTaq, 10 pM dNTPs, 5pM MgCl_2_, 10pM primers. The specific primers (sense, anti-sense) were used to amplify the specific region of *G. barbadense* annexins. The endogenous control ubiquitin gene was amplified by using primers Ub1 and Ub2 (Table S1) under the above described condition. Transcriptional changes were calculated based on the comparative _Δ_CT method [43], [48]. Each sample was repeated at least three times, and the amplification results were analyzed by Option 3 software.

### Generating transgenic *AnxGb6 Arabidopsis* plants

In order to analysis the role of *AnxGb6* gene during root and ovule development, the coding sequence of *AnxGb6* gene was cloned into *pDONR*201 vector to generate *pDONR-AnxGb6* construct. The *AnxGb6* gene was then recombined into *pBIB* vector by the Gateway LR recombination reaction (Invitrogen, CA, USA) to generate *pBIB-35S::AnxGb6::NOS* expression cassette. The construct was transferred into *Agrobacterium tumefaciens GV3101*, and then introduced into *Arabidopsis* (ecotype Columbia) plants using a floral dip method [49]. Fully-mature seeds were collected and screened on ½ MS plates containing 10mg/L glufosinate-ammonium. The germinated seedlings were transplanted into pots with a soil mixture and placed in a greenhouse for further growth. PCR was performed to verify the transgenic status of the screened plants.

The seeds of WT and transgenic *AnxGb6* lines were sterilized and grown on the ½ MS medium at 22°C under a 16 h light period. After 14 days of growth, primary root length of twenty plants from each transgenic lines and wild-type were recorded. The experiments were repeated at least four times. And the root cell length was observed by propidium iodide staining method. The roots were incubated 0.2 µM propidium iodide solution for 30 sec. Excess propidium iodide was then removed by rinsing 3 times with H_2_O. The roots were immediately examined using a confocal microscope (Leica TCS SP5). The length of root cell from the maturation zone (50 cells from WT and line L7 respectively) was counted and analyzed. The experiments were repeated at least four times.

### Sub-cellular localization of cotton annexin proteins

To investigate sub-cellular localization of cotton annexin proteins, the coding regions of cotton annexins (*AnxGb1*, *AnxGb4*, *AnxGb6*) were cloned into the *pBIB-GFP* vector to generate *pBIB-35s::AnxGb-GFP* construct. The *pBIB-35s::AnxGb-GFP* plasmid was then transformed into *Agrobacterium* strain *EHA105*. Three week-old tobacco (*Nicotiana benthamiana*) leaves were infiltrated with *Agrobacterium* [50]. Protein sub-cellular localization was analyzed 2 to 4 days after infiltration by confocal microscope (Leica TCS SP5).

### Yeast two hybridization and BiFC confirmation *in vivo*

To test proteins interaction in vitro, AnxGb6, AnxGb5, 23 calcineurin B-like calcium sensor interacting protein kinase genes (CIPK), 27 calcium-dependent protein kinase genes (CDPK), wall-associated kinase protein 1 gene and GbAct1 (AY305723) were cloned into both pGBKT7 and pGADT7 vectors. The gene sequence data of CIPKs, CDPKs and wall-associated kinase protein 1 were downloaded from the JGI web site (ftp://ftp.jgi-psf.org/pub/compgen/phytozome/v9.0/Graimondii/). Yeast two-hybrid assays were performed according to the manual of Yeast Transformation System kit (Clontech, CA, USA). Transformed AH109 yeast cells were grown on SD/-TL and incubated at 28°C for 3d. Those positive colonies were subsequently transferred to the selective and stringent, SD/-T-L-H medium or SD/-T-L-H-A supplemented with 2mM 3-AT medium.

For BiFC studies, the coding region (without a termination codon) of *AnxGb6* and *AnxGb5* were cloned into *pEarleyGate202* vector, *AnxGb6*, *AnxGb5* and *GbAct1* were cloned into *pEarleyGate201* [51]. These vectors were transformed into the *Agrobacterium* strains *EHA105* using chemical transformation. The p19 protein of tomato bushy stunt virus was used to suppress gene silencing. For co-infiltration, equal volume suspensions of different *Agrobacterium* strains carrying different constructs were mixed prior to infiltration. The re-suspended cells were infiltrated into leaves of tobacco plants as described previously.

Observation of F-actin structures in transgenic *AnxGb6 Arabidopsis* and fiber cells.

In order to investigate F-actin activity in transgenic *AnxGb6 Arabidopsis*, we generated *Arabidopsis* Col-0 expressing *CaMV35S::sgfp-ABD2-sgfp* cassette (named as WT-AC) as described previously [52]. The WT-AC homozygote with normal phenotype was then crossed with the transgenic *AnxGb6 Arabidopsis* L7 line to generate F1 hybrids. The hybrid plants were verified by PCR and used in confocal microscope observation.

To investigate the difference in fiber elongation rate among the three cotton varieties, ten ovules from each cotton variety at 3, 6, 9, 12 DPA and ten fiber cells from each ovule were used to investigate their fiber length under microscope. The experiments were repeated at least four times.

Ovules dissected from fresh bolls at 3, 6, 9 DPA were fixed in a solution of 2% paraformaldehyde in PIPES buffer (pH 6.5) for 12h. After rinsing in PBS buffer (NaCl 137mmol/L, KCl 2.7mmol/L, Na_2_HPO_4_ 10mmol/L, KH_2_PO_4_ 2mmol/L, pH 6.5), the ovules were cut into the slices of ∼1 mm thickness. Thin sections were treated with 0.05% Triton X-100 in PBS buffer for 10 min, followed by washing with PBS buffer. Finally, the sections were incubated in a solution of 0.5 µg/mol Phalloidin-TRITC (Sigma-Aldrich) in PBS buffer with 1% BSA at 37°C for 1 h. Excess phalloidin was removed by rinsing with the same buffer. The stained ovule sections were immediately examined using a confocal microscope (Leica TCS SP5).

## Results

### Identification of the *G. barbadense* annexin gene family

Plant annexins are multifunctional and structurally soluble proteins capable of calcium dependent or calcium independent membrane-binding [53]. Cotton annexins are known to associate with the cell-membrane and affect 1, 3-ß-glucan synthase activity in a calcium dependent manner [34]. Comparative proteomics of fiber elongation showed that the four cotton annexin proteins (AnxGh1: AAR13288, AnxGh2: AAB67993, AnxGhFx: FJ415173, AnxGhF: AAC33305) were more abundant in fibers of 10-dpa wild-type plants as compared with the fuzzless-lintless mutant [54]. Two-dimensional gel electrophoresis also demonstrated that the four cotton annexin iso-variants (ES793672, CO129429, ES795476 and ES804937) were down-regulated in the lintless mutant fiber [41]. These results indicate that annexins are required to sustain fiber elongation. In order to reveal the annexins' respective functions during fiber elongation period we cloned annexin genes from sea-island cotton with longer fibers.

**Figure 1:**
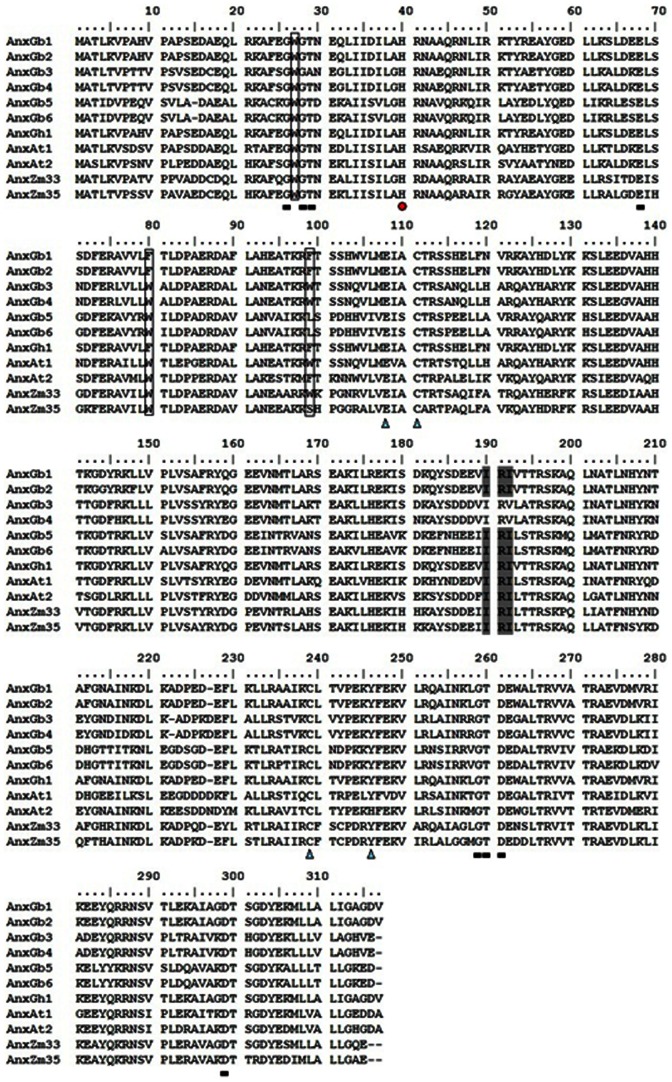
Multiple sequence alignment analysis of AnxGb1-6 and other plant annexin proteins. Potential functional domains are indicated as follows: rectangle, Calcium binding site of type II G-X-GTD-{ca. 38}-E/D; black box, conserved tryptophan required for Ca^2+^-independent membrane binding; triangle, putative S3 cluster thought to be involved in redox reactions; grey, IRI motif for binding actin; circle, conserved His residue. Amino acid sequence alignment was performed using CLUSTALW. Accession numbers are as follows: AnxAt1 (NP174810); AnxAt 2 (NP201307); AnxZm33 (NP001105728); AnxZm35 (NP001105475); AnxGh1 (AAR13288).

A total of 6 *G. barbadense* annexin genes were cloned, each of which encodes a protein that was evolutionarily conserved and similar to the structure of annexin proteins from other plants (GenBank NO.: KC316004 to KC316009). The *G. barbadense* annexins were found to contain the conserved Ca^2+^-binding sites (G-X-GTD-(ca. 38)-E/D) and four annexin repeats with 70 amino acids at their C-terminals (Figure 1). All of the six proteins contained a heme binding motif of 30 amino acids, which contained the conserved His residue for heme binding similar to that in peroxidase from *Armoracia rusticana* [55]. The cotton proteins also contained S3 clusters putatively involved in redox reactions [56]. Interestingly, a potential F-actin binding motif (IRI) was found in AnxGb5 and 6, while AnxGb3 and 4 contained IRV amino acid residues at the same site [57].

The deduced annexin protein sequences were used to predict their putative signal peptides, protein lengths, molecular masses, and pI values on the ExPASy website. The results showed that none of the 6 AnxGbs contained putative signal peptides. The annexin proteins were predicted to contain 316 (AnxGb1 and 2), 315 (AnxGb3 and 4), and 314 (AnxGb5 and 6) amino acids respectively, corresponding to molecular masses of 36.06 to 35.80 kDa. AnxGb1–6 proteins were predicted to have pIs in the acidic range (6.19–6.74). Based on the different protein structures, MW and pI we could divide all of the 6 annexins into 3 groups: 1) AnxGb1 and 2; 2) AnxGb3 and 4; and 3) AnxGb5 and 6.

**Figure 2:**
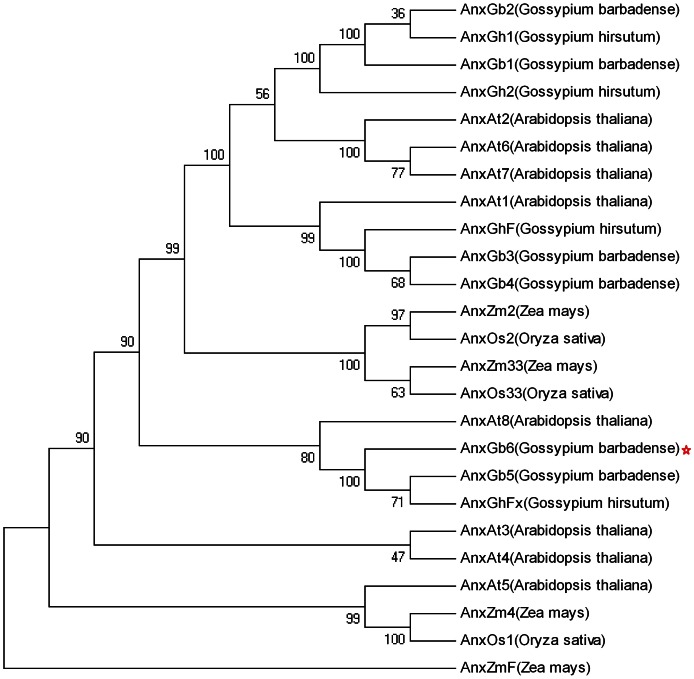
Phylogenetic analysis of *G. barbadense* annexins. Nineteen of the complete amino acid sequences of annexin proteins were used to generate the Neighbor–Joining tree, and the numbers next to each node give bootstrap values from 1000 replicates.(AnxGh1: AAR13288.1; AnxGh2: AAB67994.1; AnxGhF: AAC33305.1; AnxGhFx: FJ415173; AnxAt1: NP_174810.1; AnxAt2: NP_201307.1; AnxAt3: NP_181410.1; AnxAt4: NP_181409.1; AnxAt5: NP_564920.1; AnxAt6: NP_196584.1; AnxAt7: NP_196585.1; AnxAt8: NP_568271.2; AnxZm2: NP_001105475.1; AnxZm4: NP_001147343.1; AnxZmF: ACF82214.1; AnxZm33: NP_001105728.1; AnxOs1: NP_001061839.1; AnxOs2: NP_001048149.1; AnxOs33: NP_001057176.1)

In order to look more closely at the relationships between *G.barbadense* annexins and the other members of the plant annexin protein family, the multiple alignments of full-length protein sequences were used to construct a Neighbor-Joining phylogenetic tree. The phylogenetic tree includes 6 *G. barbadense* annexin proteins, AnxGh1, AnxGh2, AnxGhF and AnxGhFx in cotton; AnxAt1-8 in *Arabidopsis*; AnxZm2, AnxZm4, AnxZmF and AnxZm33 in Maize; AnxOs1, AnxOs2 and AnxOs33 in Rice (Figure 2). Phylogenetic analysis revealed that 6 *G. barbadense* annexins genes were classified into 3 groups, which was consistent with the alignment result. The deduced amino acid sequences for AnxGb5 and 6, have predicted actin-binding sites, while the other *G. barbadense* annexins do not, indicating that they may have a distinct function in cotton fiber development. Therefore, we used expression pattern analysis and sub-cellular localization analysis to verify these inferences.

### *AnxGb6* is predominantly expressed in the elongation fiber

**Figure 3:**
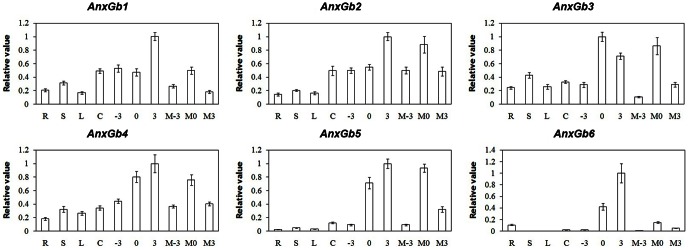
Real-time quantitative PCR analysis of the annexin genes in Pima-90 and XU142 fl. Expression analysis of annexin genes in *G. barbadense* (Pima-90) vegetative tissues (R: roots; S: stems; L: leaves), reproductive tissues (C: carpels; −3: ovules in –3 DPA; 0: ovules in 0 DPA; 3: ovules in +3 DPA) and its allele gene expression in *G. hirsutum* fuzzless-lintless mutant (XU142 fl) reproductive tissues (M −3; ovules in –3 DPA; M0: ovules in 0 DPA; M3: ovules in +3 DPA). The comparative C_T_ method was adopted and the expression was normalized to the levels of Pima-90 and XU142 fl. Error bars represent standard errors.

Real-time quantitative PCR (qRT-PCR) was performed to investigate the spatial expression patterns of *G. barbadense* annexin genes in cotton (Figure 3, Figure S1). The qRT-PCR results showed that the expression of all annexin genes was higher in the reproductive tissues than in the vegetative tissues. *AnxGb5* and *6* were mainly expressed in the ovule tissues, while very low signals were detected in vegetative tissues (*AnxGb6* expression in root), indicating that the functions of *AnxGb5* and *6* genes were related to fiber development. In order to get more accurate data for expression pattern of annexins during fiber initials, we performed qRT-PCR to analyze their alleles in *G. hirsutum* fuzzless-lintless mutant. *AnxGb5* and *6* genes had similar expression patterns during fiber initiation, their expression levels were found to constantly increase in the ovules from -3 DPA to 3 DPA. Interestingly, the expression of all 6 annexin genes was found to be significantly higher in the developing fiber cells of Pima-90 in comparison with that of XU142 fiberless mutant, implying a general role for annexins during fiber development and growth (Figure S1).

### Cotton annexin proteins are localized in the plasma membrane and nucleolus

**Figure 4:**
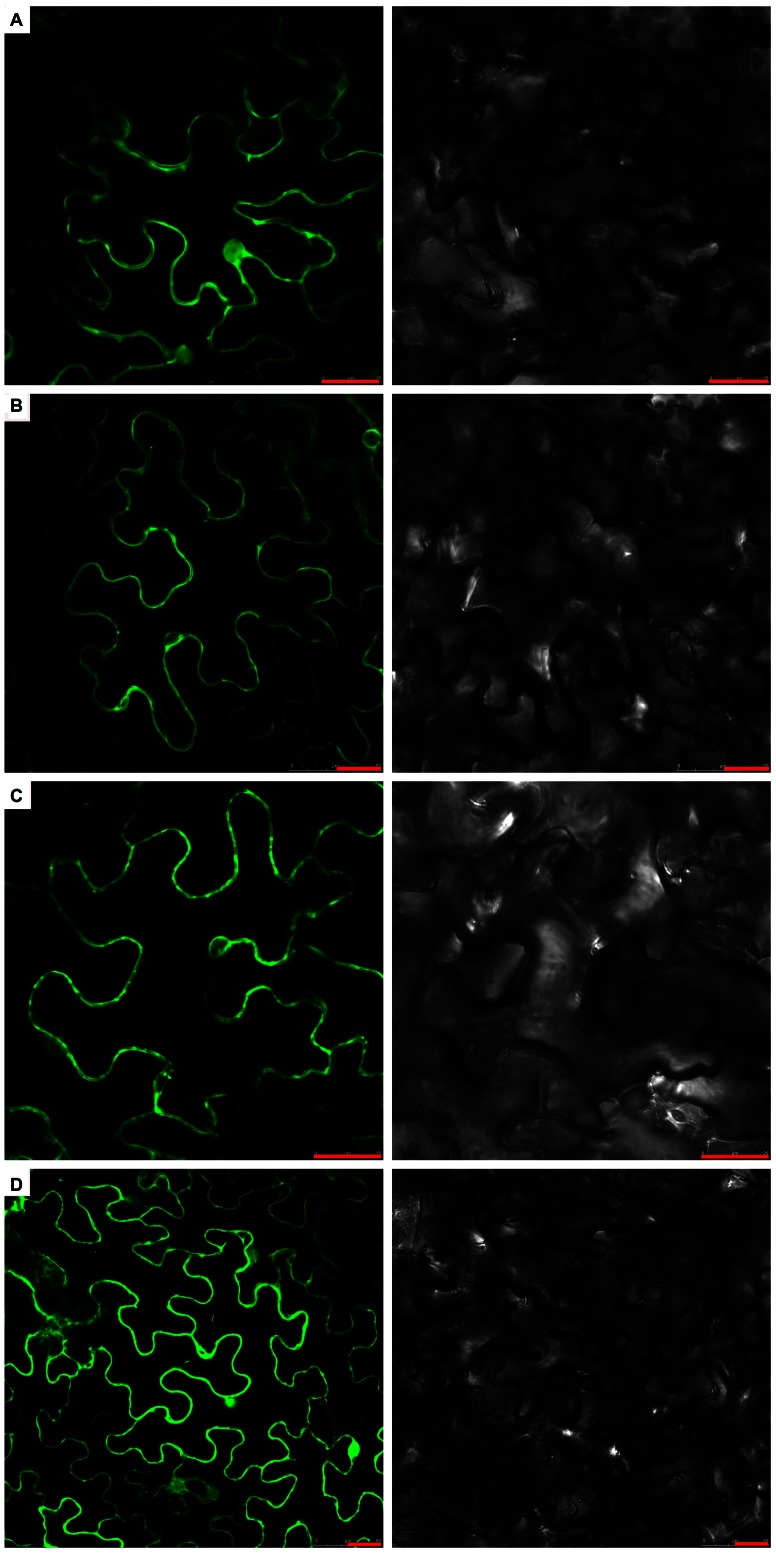
Cotton annexin protein sub-cellular localization analysis. A: AnxGb1-GFP, B: AnxGb4-GFP, C: AnxGb6-GFP and D: Control plants, expressing 35S::GFP. Right is the corresponding bright-field. Left is the corresponding black-field. Scale bar: 25 µm.

In order to investigate the sub-cellular location of *G. barbadense* annexins, three genes *AnxGb1*, *4*, *6* from different cotton annexin groups were chosen to generate GFP fusion protein constructs (*pBIB-35S::AnxGb1*, *4*, *6-GFP*). The constructs were introduced into *Agrobacterium EHA105* and infiltrated into tobacco leaf cells. Confocal microscopy showed that the three annexins, *AnxGb1*, *4*, *6* had the same sub-cellular location, and had a high expression in the plasma membrane and nucleolus (Figure 4).

### Ectopically expressed *AnxGb6* gene in *Arabidopsis* enhanced its root elongation

**Figure 5:**
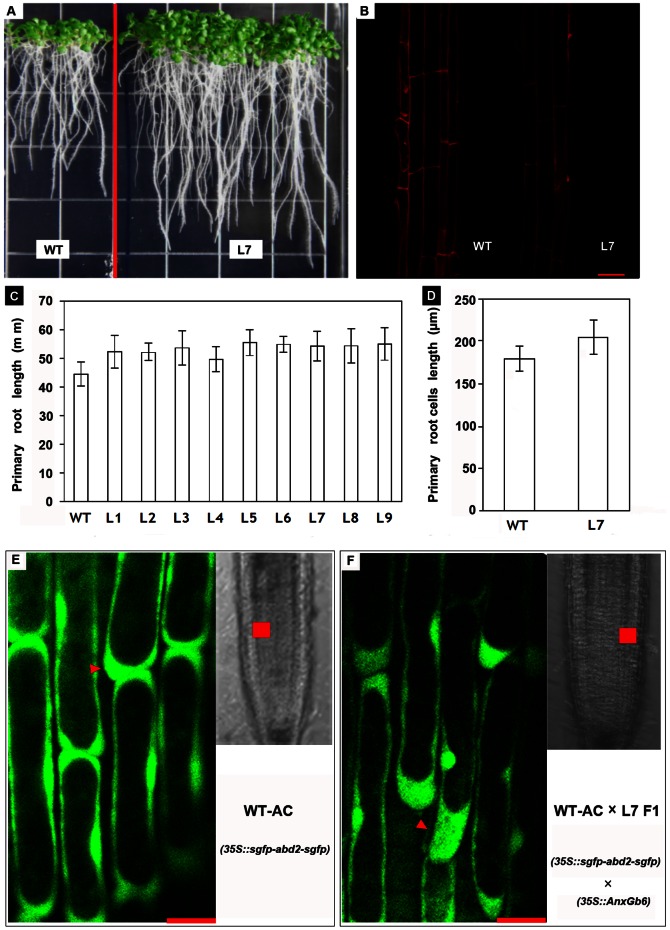
Phenotypes of transgenic *AnxGb6 Arabidopsis* plants. A: *Arabidopsis* seedlings grown in ½ MS media for 14 days. (WT: Wild type; L7: transgenic *AnxGb6* line L7). B: Confocal images of primary roots cells stained with propidium iodide. (WT: Wild type; L7: 35S::*AnxGb6* transgenic line L7; Scale bar: 50 µm). C: Morphometric analysis of the transgenic *Arabidopsis* plants root growth for 14 days (WT: Wild type; L1–L9: 35S::*AnxGb6* transgenic lines; Error bars represent standard errors). D: Morphometric analysis of the transgenic *Arabidopsis* plant root cell length (WT: Wild type; L7: 35S::*AnxGb6* transgenic line L7; Error bars represent standard errors). E–F: Confocal images of F-actin accumulation in *Arabidopsis* primary roots cells. E: Wild type *Arabidopsis* roots cells, Scale bar: 12.5 µm. F: Transgenic *Arabidopsis* roots cells, Scale bar: 12.5 µm.

In order to gain further insight into the *AnxGb6* function, *AnxGb6* gene was ectopically expressed in *Arabidopsis*. Nine independent transgenic *AnxGb6* lines were obtained. Since *AnxGb6* gene was also found to be expressed at very low levels in the root, we investigated the effect of *AnxGb6* over-expression on root growth. The root growth of all transgenic *AnxGb6* lines was found to be enhanced as compared to the control plants (Figure 5A). However, the number and formation process of lateral and adventitious roots did not show obvious differences between transgenic and wild type plants. Transgenic *AnxGb6* seedlings had much longer primary roots after 14-days on ½ MS media, and their elongation rates were 11.5 to 24.7% greater than that of wild-type (Figure 5C, Table S2).

To detect how *AnxGb6* affected the root elongation in *Arabidopsis*, we chose line L7 with high *AnxGb6* gene expression to investigate the morphological changes in root cells. Propidium iodide was used to stain cell walls of transgenic and wild-type plants. Microscopic analysis revealed that the number of cells in both apical meristem and elongation zones did not differ significantly between L7 and wild type plants (data not shown). The increase in root length in transgenic lines was due to the enlargement of cell size in the longitudinal direction. The cell length of L7 plants was 10 to 23.6% longer than that of wild-type seedlings in the primary root zone (Figure 5B, D).

### AnxGb6 specifically interacts with GbAct1 during fiber elongation

**Figure 6:**
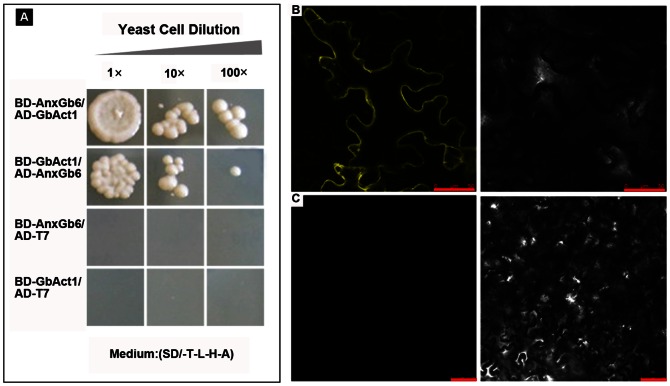
AnxGb6 interacts with GbAct1. A: AnxGb6 binds to GbAct1. Yeast harboring BD-AnxGb6/AD-GbAct1 or BD-GbAct1/AD-AnxGb6 grown on selective plates as indicated. Control medium: (SD/-T-L-H-A) selective medium. The control is yeast transformed with BD-AnxGb6/ADT7 and BD-GbAct1/ADT7. Dilution multiple from left to right is 1 fold, 10 fold, 100 fold. B–C: BiFC of epidermal cells co-expressing split YFP fusions of AnxGb6 and GbAct1 or empty vector controls. Combinations of N- and C-terminal YFP fragments (*Yn* and *Yc*, respectively) were infiltrated as vector controls or fused to the N terminus of AnxGb6 and GbAct1 as follows: B: AnxGb6-*Yc* and GbAct1-*Yn*, Scale bar: 50 µm; C: vector-*Yc* and vector-*Yn*, Scale bar: 100 µm. The interaction and co-localization were observed at the plasma membrane. Right is the corresponding bright-field.

Recent studies have shown that animal annexins can interact with several protein kinases and F-actin to regulate membrane trafficking and actin reorganization [58], [59]. In plants, protein pull-down analysis showed that a rice annexin protein Os05g31750 probably interacted with Ste20-like kinase Os10g37480, SPK-3 kinase Os01g64970 and casein kinase Os01g28950 [60]. F-actin affinity and chromatography experiments provided evidence that tomato and *Mimosa* annexins had F-actin binding activity *in vitro* [61]–[64]. However, systemic investigation of the involvement of annexin proteins in fiber development has not been demonstrated. Since annexins are important regulators of membrane trafficking in animal and are known to influence polar growth in *Arabidopsis* [59], [65], we predicted that annexins AnxGb6 protein probably had a similar function during fiber elongation in cotton. Therefore, we used AnxGb6 protein as bait to test its interaction with potential protein candidates including 23 CIPKs, 27 CDPKs, wall-associated kinase protein 1 and GbAct1 [10]. Yeast two hybridization results showed that AnxGb6 did not interact with CIPKs, CDPKs, and wall-associated kinase proteins (data not shown). Yeast cells co-transformed with BD-AnxGb6 and AD-GbAct1 could grow on the selective medium SD-Leu-Ade-Trp-His with 2 mM 3-AT, while the control group (AD and BD-AnxGb6) and (AD and BD-GbAct1) could not grow (Figure 6A). These results indicated that AnxGb6 could directly bind to GbAct1 instead of wall-associated kinase1, CIPK and CDPK family proteins in cotton.

To confirm their proteins interactions *in vivo*, we used bimolecular fluorescence complementation (BiFC) to identify the interaction localization in tobacco leaf cells. The BiFC assay is based on the formation of a fluorescent complex comprising of two fragments of YFP, which are brought together by the association of two interacting proteins fused to the YFP N/C terminals [66]. When GbAct1-N-terminal YFP was co-infiltrated with AnxGb6-C-terminal YFP, fluorescence was observed in the plasma membrane (Figure 6B). The BiFC experiments demonstrated that AnxGb6 and GbAct1 actually interacted in plants and as predicted, they are localized in the plasma membrane.

**Figure 7:**
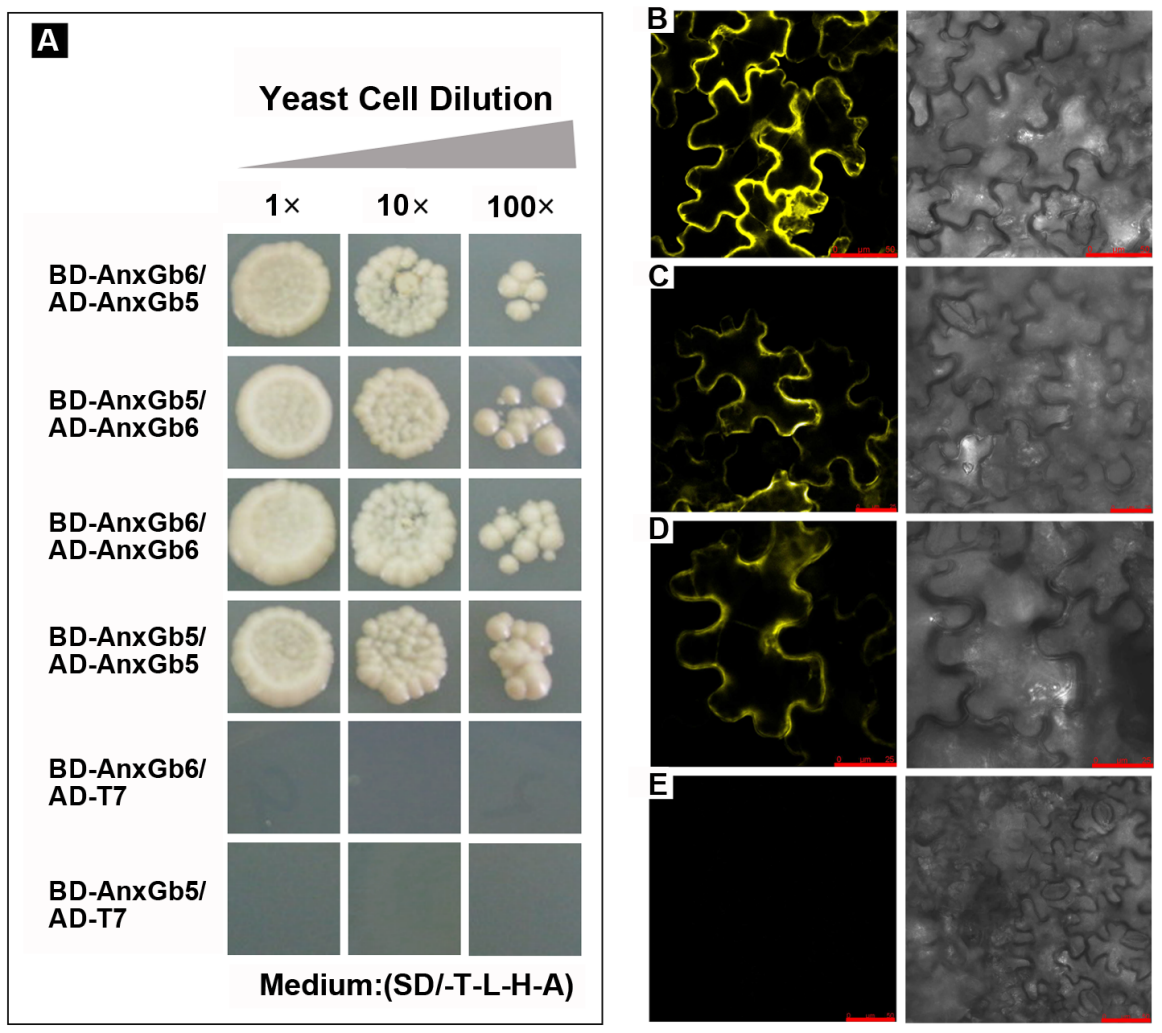
AnxGb5 and 6 interact with each other and self-associate. A: AnxGb6 binds AnxGb5 or itself. Yeast harbouring BD-AnxGb6/AD-AnxGb5, BD-AnxGb5/AD-AnxGb6, BD-AnxGb6/AD-AnxGb6 and BD-AnxGb5/AD-AnxGb5 grown on selective plates as indicated. Control medium: (SD/-T-L-H-A) selective medium. The control yeast transformed with BD-AnxGb6/ADT7 and BD-AnxGb5/ADT7. Dilution multiple from left to right is 1 fold, 10 fold, 100 fold. B-E: BiFC of epidermal cells co-expressing split YFP fusions of AnxGb6 and AnxGb5 or empty vector controls. Combinations of N- and C-terminal YFP fragments (*Yn* and *Yc*, respectively) were infiltrated as vector controls or fused to the N terminus of AnxGb6 and AnxGb5 as follows: B: AnxGb6-*Yc* and AnxGb5-*Yn*, Scale bar: 50 µm; C: AnxGb6-*Yc* and AnxGb6-*Yn*, Scale bar: 25 µm; D: AnxGb5-*Yc* and AnxGb5-*Yn*, Scale bar: 25 µm; E: vector-*Yc* and vector-*Yn*, Scale bar: 50 µm. The interaction and co-localization was observed in the plasma membrane. Right is the corresponding bright-field.

In animals, annexin-annexin protein interactions are able to provide a stable cytoskeleton with bisphosphate protein, resulting in the formation of a protein scaffold with subsequent F-actin recruitment [67]. Thus we hypothesized that AnxGb5 and 6 could interact with GbAct1, and also interact with themselves to generate a protein complex scaffold. In order to validate this speculation, yeast two hybridization and BiFC were performed. As shown in Figure 7, both AnxGb5 and AnxGb6 could interact with themselves to form a homodimer complex (AnxGb5-AnxGb5, AnxGb6-AnxGb6) (Figure 7A, C, D). AnxGb5 was also found to be capable of binding with AnxGb6 to form a heterodimer complex (Figure 7A, B). Unlike AnxGb6, AnxGb5 could not directly bind with GbAct1 protein *in vitro* (data not shown). In conclusion, the annexin proteins in subgroup III could assemble a membrane targeting protein complex, which provided a domain for AnxGb6 homodimer to directly bind with GbAct1 protein for actin polymerization.

### *AnxGb6* expression correlates with F-actin activities and fiber elongation

To confirm whether higher *AnxGb6* expression could result in F-actin changes in *Arabidopsis*, we used F-actin specific combining polypeptide and SGFP fusion protein to mark F-actin distribution in transgenic *AnxGb6* plants [68]. As shown in Figure 5 E–F, F-actin distribution was different in transgenic and control plants. In wild type or control plants, F-actin signals could be seen in the membrane and cytoplasm. In transgenic plants, F-actins aggregated more densely in the basal tip elongation zone than in the wild type. This aggregation, which was oriented in the direction of root growth also accounted for the role of AnxGb6 in enhancing root length.

**Figure 8:**
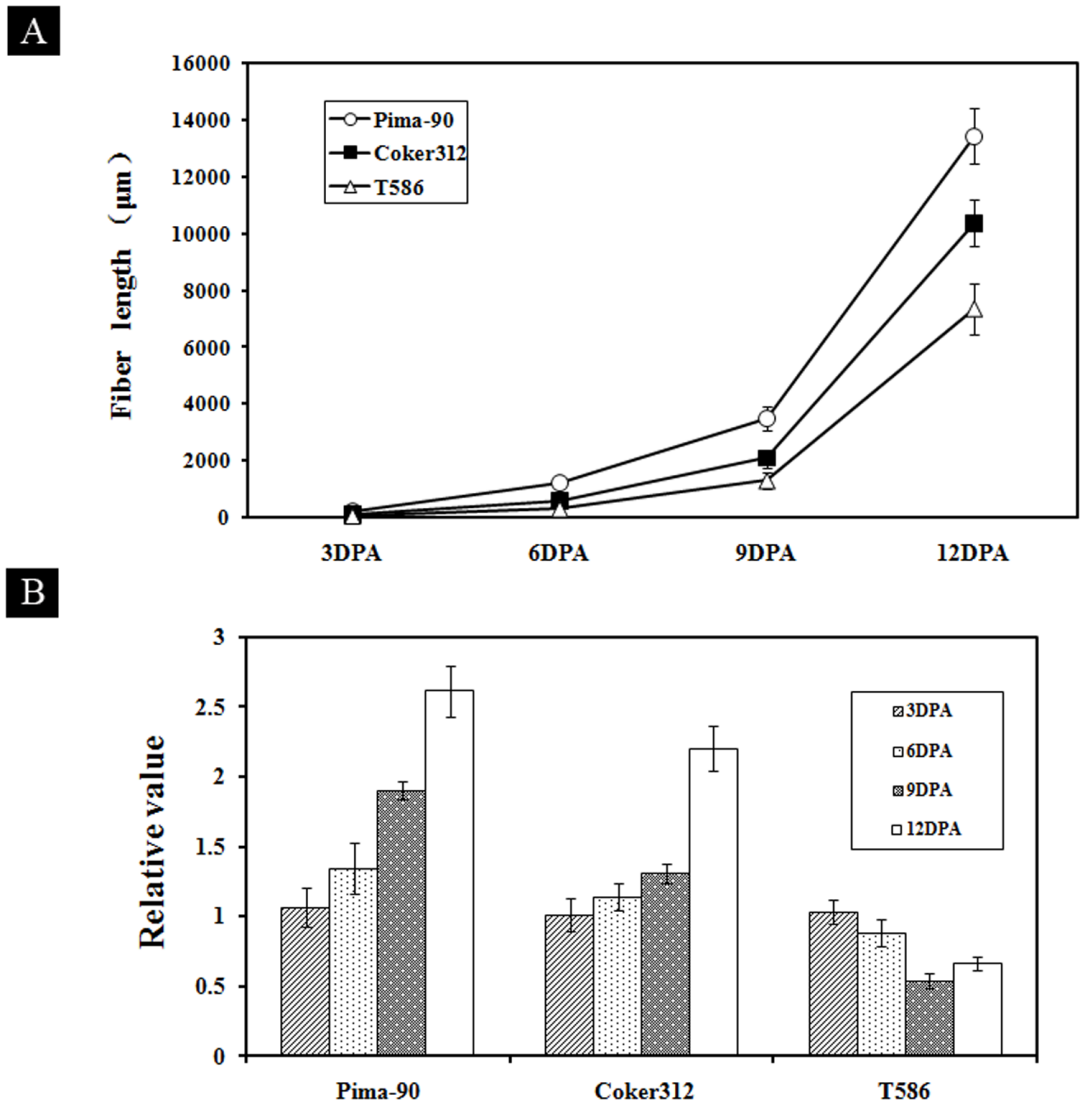
Fiber length and *AnxGb6* gene expression pattern in Pima-90, Coker312 and T586 cotton seeds. A: Fiber length of Pima-90, Coker312 and T586 cotton seeds at +3, +6, +9 and +12 DPA. Ovules were sectioned and the length of 400 fiber cells was measured under a microscope for each type. Data was processed with Microsoft Excel. Error bars represent standard errors. B: Real-time quantitative PCR analysis of the *AnxGb6* gene and its alleles in Pima-90, Coker312 and T586. Expression analysis of *AnxGb6* gene and its alleles in Pima-90 (*G. barbadense* L.), Coker312 (*G. hirsutum* L.) and T586 (*G. hirsutum* L.). 3DPA: ovules in +3 DPA, 6DPA: ovules in +6 DPA, 9DPA: ovules in +9 DPA, 12DPA: ovules in +12DPA. The comparative C_T_ method was adopted and the expression was normalized to the levels of Pima-90, Coker312 and T586. Error bars represent standard errors.

To characterize the effect of *AnxGb6* expression on fiber elongation, we chose three cotton varieties with different fiber length including Pima-90 (*G. barbadense*), Coker312 (*G. hirsutum*) and T586 (*G. hirsutum*) for fiber elongation activity analysis. The average fiber lengths of Pima-90, Coker312 and T586 were 33.04±0.28mm, 28.76±0.17mm and 21.09±0.41mm respectively in Shanghai during 2010–2012. At 1 DPA, the fiber cells differentiated and rapidly emerged from the seed-coat surface in all the three cotton cultivars. Thereafter, there was a stage of quick elongation in fiber length (Figure 8A, Table S3). The growth rate of all three cotton varieties was found increased rapidly between 9 and 12 DPA. At 6 DPA, the fiber cells in Pima-90 plants were about 1210 µm long. Six days later (12 DPA), they reached ∼13432 µm in Pima-90 (Figure 8A). The comparison of elongation rates in the 3 tested varieties demonstrated that Pima-90 had the fastest elongation rate followed by Coker 312 and T586. Fiber length of T586 at 6 DPA was only about 200–300 µm, which was equal to fiber length in Pima-90 at 3 DPA. Fiber elongation rate in T586 was 1.5 and 3 fold slower than that in Coker312 and Pima-90 respectively. Previous studies indicate that the elongation time is not significantly different for most of varieties, which is about 20 days for different cotton germplasms with different fiber lengths [69]. Therefore, our results suggested that fiber length was mostly determined by elongation rate and not elongation time.

During fiber elongation from 3 DPA to 12 DPA, there were significant differences in the expression of *AnxGb6* and its alleles (Figure 8B). Firstly, the expression level of *AnxGb6* gene in Pima-90 was highest followed by Coker312, and was the lowest in T586. Secondly, the time scale of sustained increase in *AnxGb6* gene expression was different. On comparison of *AnxGb6* gene expression patterns (from 3 to 12 DPA) among T586, Coker 312, and Pima-90 we found that the highest expression level in Pima-90 and Coker312 occurred at 12 DPA, and occurred 9 days later in T586. Time-course of the constantly increasing expression of *AnxGb6* in Pima-90 and Coker312 was found to be longer than that in T586 (Figure 8B). These results led us to infer that *AnxGb6* gene expression levels were correlated to their fiber length and elongation rates.

**Figure 9:**
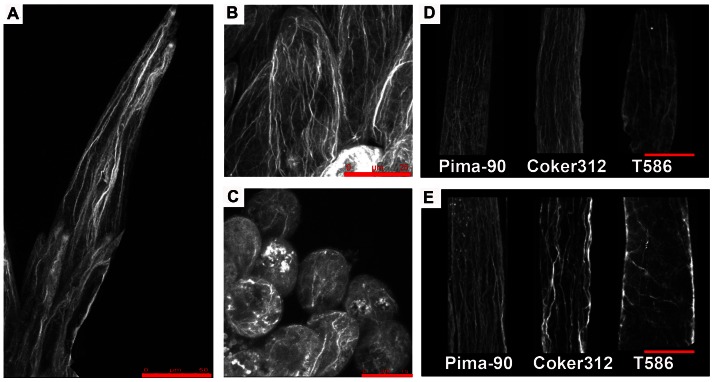
Comparison of F-actin organization in fiber cells between Pima-90, Coker312 and T586 Plants. A-C: Fiber cells at +3 DPA. A: Pima-90, B: Coker312, and C: T586. Note the length of fiber is different in the three varieties at the same stages. D: Fiber cells at +6 DPA and E: Fiber cells at +9 DPA. Bars: 10 µm in D and E.

Phalloidin staining showed that the three cotton varieties had different amounts of F-actin during fiber elongation phase (Figure 9). At 3 DPA, the bright F-actin complex was observed in the tip zones of expanding fiber cells. Flamentous F-actin was continuous from the tip of fiber cell to the base of seed coat surface in Pima-90 (Figure 9A). At 6 DPA, the actin filaments of Pima-90 and Coker312 formed cables that were arrayed parallel to the axis of the fiber elongation, while F-actin organization in the T586 fiber cells were monomeric (Figure 9D). At 9 DPA, Pima-90 and Coker312 fibers had a larger number of filamentous F-actin fibers than T586 (Figure 9E). The reduction of actin protein level correlated with the shorter fiber length. This result was consistent with the *AnxGb6* expression pattern during fiber development (Figure 8B).

## Discussion

Annexins are a family of membrane binding proteins found in both plants and animals. They are homologous, structurally related proteins known to have membrane associations including secretion, construction of ion channels, and cytoskeletal interactions [22]–[29]. Several studies investigating the differential expression of annexins between wild type and fiber length mutants indicated that cotton annexins may play an important role in fiber development [39]–[41], [54]. However, direct evidence linking any of the annexins in fiber elongation was lacking, furthermore, the function of annexins, specifically expressed in the fibers, remained unclear. In this study, we demonstrated that an annexin subfamily directly participated in cotton fiber elongation by interacting with a fiber-specific protein GbAct1. Our study filled a major gap in the understanding of protein interactions that link the membrane of fiber cells to actin reorganization during fiber elongation. In addition, we provide evidence that the formation of an annexin interaction complex influences fiber length by regulating the level of filamentous actin.

### AnxGb6 specifically participates in rapid fiber elongation

Annexins are encoded by a multi-gene family that comprises dozens of annexin genes in eukaryotes [53]. Genome-wide search revealed that at least 26 annexin genes exist in the cotton genome [70], [71]. Proteomic analysis indicated that about 4 annexins are differentially expressed in fiber elongation [41]. It is commonly known that fiber development stages overlap with embryo development and seed maturation. The annexin member involved in fiber cell expansion rather than embryo enlargement and seed maturation needs to be determined. Therefore, we cloned all of the *annexin* genes abundantly expressed in the fibers at the genome wide level and confirmed that AnxGb6 specifically participated in rapid fiber elongation based on our results.

*AnxGb1-5* was found to be highly expressed in the ovule and developing fiber; however, *AnxGb6* was predominantly expressed in the developing fiber of sea-island cotton and was barely detectable in the ovule of the fiber-less mutant (Figure 3). The ectopic expression of *AnxGb6* in *Arabidopsis* led to the formation of longer roots further reiterating the involvement of *AnxGb6* in cell elongation (Figure 5A). Propidium iodide staining of the cell wall confirmed that longer roots resulted from cell elongation rather than increase in the number of cells (Figure 5B). The actin marker clearly indicated that more actin aggregation appeared in the root elongation zone of transgenic plants. It was found that actins aggregated at the base of root cells consistent with the direction of root elongation (Figure 5F). Moreover, the difference in expression of *AnxGb6* among the three genotypes Pima-90, Coker 312 and T586 with different fiber lengths confirmed that *AnxGb6* was correlated to fiber elongation (Figure 8). These results strongly support the fact that AnxGb6 is involved in the cell polarity elongation in fiber. These findings also imply that there is a close interplay between higher expression of AnxGb6 and increased actin aggregation.

### AnxGb6 promotes fiber elongation most likely through regulating its expanding rate

Previous studies have debated whether actin filament cables are responsible for delivering the cellulose synthase-containing vesicles into the plasma membrane [72]. Actin 1 has been proven to be a pivotal factor in fiber elongation [10]. Therefore, finding the protein that interacts with actin 1 would help explain the mechanism of fiber elongation.

Our study showed that AnxGb6 could interact with GbAct1 *in vitro* as well as *in vivo* (Figure 6). The increased *AnxGb6* expression resulted in F-actins that aggregated in the direction of root growth, indicating that *AnxGb6* contributed to polar cell expansion (Figure 5F). Comparative analysis of *AnxGb6* expression in different genotypes showed that lower *AnxGb6* expression caused the formation of shorter fibers and accumulation of a lower amount of actin in expanding cells (Figure 8B, 9). This result was consistent with a previous study that AnxGb6 proteins were markedly down-regulated in the shorter fiber length *li* mutants [41]. Furthermore, ectopic expression of a mustard annexin gene *AnnBj1* in cotton plants is known to increase the fiber length [42]. These studies indicate that *AnxGb6* expression helps accelerate actin bundle organization and also affects fiber elongation. Comparative analysis of fiber length and elongation rate in different cotton species determined that fiber elongation rate was the most important factor in determining fiber length in domesticated cotton species [69]. Hence, *AnxGb6* expression could possibly improve actin bundle architecture and influence fiber elongation rate (Figure 8, 9). Thus, *AnxGb6*, a member of the annexin superfamily of proteins, through its interactions with actin 1 regulates the elongation of cotton fibers. Further study is needed to enhance our understanding of the mechanism by which *AnxGb6* influences the fiber extension rate.

## Supporting Information

Figure S1 **Quantitative RT-PCR analysis of the annexin genes in parallel growth stages of Pima-90 and XU142fl.** Expression analysis of annexin genes in *G. barbadense* (Pima-90) and its allele gene expression in *G. hirsutum* fuzzless-lintless mutant (XU142 fl) reproductive tissues (-3 DPA: ovules in –3 DPA; 0 DPA: ovules in 0 DPA; 3 DPA: ovules in +3 DPA). The comparative C_T_ method was adopted and the expression was normalized to the levels of Pima-90 and XU142fl. Error bars represent standard errors. Statistical significance between the pair tested material was determined using Student's t-test; ***, significant at p < 0.001.
(TIF)

Table S1 **Primers used in this study.**
(DOCX)

Table S2 **Root length of the transgenic** ***AnxGb6*** **and wild type** ***Arabidopsis*** **plants after growth in the ½ MS for 14 days.**
(DOCX)

Table S3 **Fiber length in Pima-90, Coker312 and T586 during fiber elongation stage (0-12DPA).**
(DOCX)
