# Supplementary figures and images for: A Cotton Annexin Protein AnxGb6 Regulates Fiber Elongation through Its Interaction with Actin 1
Source: PLoS One. 2013 Jun 4;8(6):e66160. doi: 10.1371/journal.pone.0066160 (PMC3672135; doi:10.1371/journal.pone.0066160)

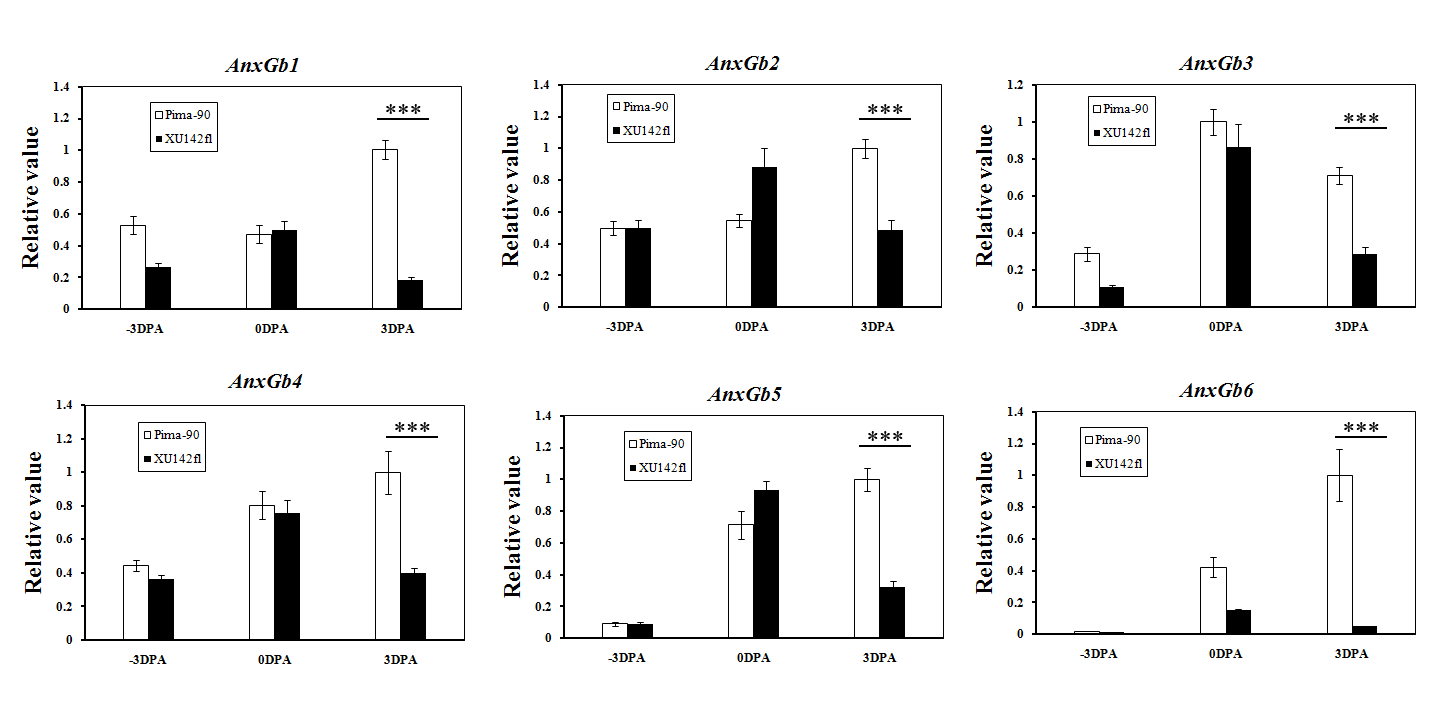

Supplement: Figure S1 — Quantitative RT-PCR analysis of the annexin genes in parallel growth stages of Pima-90 and XU142fl. Expression analysis of annexin genes in G. barbadense (Pima-90) and its allele gene expression in G. hirsutum fuzzless-lintless mutant (XU142 fl) reproductive tissues (-3 DPA: ovules in –3 DPA; 0 DPA: ovules in 0 DPA; 3 DPA: ovules in +3 DPA). The comparative CT method was adopted and the expression was normalized to the levels of Pima-90 and XU142fl. Error bars represent standard errors. Statistical significance between the pair tested material was determined using Student's t-test; ***, significant at p < 0.001. (TIF) [file pone.0066160.s001.tif]
