# Supplementary material for: A Cotton Annexin Protein AnxGb6 Regulates Fiber Elongation through Its Interaction with Actin 1
Source: PLoS One. 2013 Jun 4;8(6):e66160. doi: 10.1371/journal.pone.0066160 (PMC3672135; doi:10.1371/journal.pone.0066160)
Supplement: Table S1 — Primers used in this study. (DOCX) [file pone.0066160.s002.docx]

**Table S1**: Primers used in this study.

| Primers | Sequence 5′–3′ |
| --- | --- |
| An1F | ATGGCAACCCTTAAAGTTCCAGCT for *AnxGb1, 2* ORF cloning |
| An1R | TCAGACGTCTCCAGCTCCAATCAA for *AnxGb1, 2* ORF cloning |
| An2F | ATGGCCACTCTTACAGTGCCCACG for *AnxGb3, 4*ORF cloning |
| An2R | TCACTCCACATGTCCTGCAAGTAC for *AnxGb3, 4*ORF cloning |
| An3F | ATGGCCACCATTGATGTTCCTGAG for *AnxGb5, 6*ORF cloning |
| An3R | TCAATCTTCTTTTCCCAACAGAGT for *AnxGb5, 6*ORF cloning |
| rAN1F | GAGACTACCGCAAGCTTTTGGTC for *AnxGb1* qRT-PCR |
| rAN1R | CTCAGCAATTTGAGGAATTCGTCTT for *AnxGb1* qRT-PCR |
| rAN2F | TACCAAGGGAGGCTACCGCAAGT for *AnxGb2* qRT-PCR |
| rAN2R | GCAATTTCAGGAATTCGTCTTCG for *AnxGb2* qRT-PCR |
| rAN3F | GTCACAGAAATGCCGAGCAG for *AnxGb3* qRT-PCR |
| rAN3R | GCAGGCTATTTCCATAAGGACC for *AnxGb3* qRT-PCR |
| rAN4F | TAAGAAGTCGCTTGAAGAGGG for *AnxGb4* qRT-PCR |
| rAN4R | GTCAGCCTTCAAGTCCTTGTC for *AnxGb4* qRT-PCR |
| rAN5F | TTGCGTGCGACGATTCGATGCCTC for *AnxGb5* qRT-PCR |
| rAN5R | GAAGCAATGCCTTGTAATCCCCTGA for *AnxGb5* qRT-PCR |
| rAN6F | TGATTTCGAGGAAGCAGTGTATCG for *AnxGb6* qRT-PCR |
| rAN6R | TTCAACGATCACATGGTGATCAGG for *AnxGb6* qRT-PCR |
| Ub-1 | AAGACCTACACCAAGCCCAA, for qRT-PCR |
| Ub-2 | AAGTGAGCCCACACTTACCA, for qRT-PCR |
