# Supplementary material for: A Cotton Annexin Protein AnxGb6 Regulates Fiber Elongation through Its Interaction with Actin 1
Source: PLoS One. 2013 Jun 4;8(6):e66160. doi: 10.1371/journal.pone.0066160 (PMC3672135; doi:10.1371/journal.pone.0066160)
Supplement: Table S2 — Root length of the transgenic AnxGb6 and wild type Arabidopsis plants after growth in the ½ MS for 14 days. (DOCX) [file pone.0066160.s003.docx]

**Table S2**: Root length of the transgenic *AnxGb6* and wild type *Arabidopsis* plants after growth in the ½ MS for 14 days.

| Arabidopsis lines | Root length (cm) |
| --- | --- |
| WT(wild type) | 44.48±4.21 |
| L1 (transgenic line) | 52.25±5.75 |
| L2 (transgenic line) | 52.03±3.43 |
| L3 (transgenic line) | 53.60±6.02 |
| L4 (transgenic line) | 49.64±4.36 |
| L5 (transgenic line) | 55.47±4.53 |
| L6 (transgenic line) | 54.85±2.79 |
| L7 (transgenic line) | 54.21±5.15 |
| L8 (transgenic line) | 54.31±5.98 |
| L9 (transgenic line) | 55.06±5.69 |

Note: Primary root length of twenty plants from each transgenic lines and wild-type were investigated after 14 days growth in the ½ MS (at 22 ℃ under a 16 h light period). The experiments were repeated at least four times.
