# Supplementary material for: A Cotton Annexin Protein AnxGb6 Regulates Fiber Elongation through Its Interaction with Actin 1
Source: PLoS One. 2013 Jun 4;8(6):e66160. doi: 10.1371/journal.pone.0066160 (PMC3672135; doi:10.1371/journal.pone.0066160)
Supplement: Table S3 — Fiber length in Pima-90, Coker312 and T586 during fiber elongation stage (0-12DPA). (DOCX) [file pone.0066160.s004.docx]

**Table S3**: Fiber length in Pima-90, Coker312 and T586 during fiber elongation stage (0-12DPA).

| Cotton varieties | Day post anthesis | Fiber length (μm) |
| --- | --- | --- |
| Pima-90 | 3DPA | 202.08±20.03 |
|  | 6DPA | 1210.37±143.64 |
|  | 9DPA | 3500.20±423. 06 |
|  | 12DPA | 13432.94±989.13 |
| Coker312 | 3DPA | 86.10±19. 83 |
|  | 6DPA | 580. 76±103.63 |
|  | 9DPA | 2080. 00±315.38 |
|  | 12DPA | 10370.29±808.04 |
| T586 | 3DPA | 31.26±10.55 |
|  | 6DPA | 276.19±85.94 |
|  | 9DPA | 1280.35±284.08 |
|  | 12DPA | 7329.38±889.66 |

Note: Ten ovules from each cotton varieties at 3, 6, 9, 12 DPA and ten fiber cells from each ovule were used to investigate their fiber length under microscope. The experiments were repeated at least four times.
